# Zerumbone-Loaded Nanostructured Lipid Carrier Gel Enhances Wound Healing in Diabetic Rats

**DOI:** 10.1155/2022/1129297

**Published:** 2022-09-10

**Authors:** Shaymaa Fadhel Abbas Albaayit, Rasedee Abdullah, Mohd Hezmee Mohd Noor

**Affiliations:** ^1^Department of Biology, College of Science, University of Baghdad, Baghdad, Iraq; ^2^Institute of Biological Sciences, Faculty of Science, University of Malaya, 50603 Kuala Lumpur, Malaysia; ^3^Department of Veterinary Laboratory Diagnosis, Faculty of Veterinary Medicine, Universiti Putra Malaysia, 43400 Serdang, Selangor, Malaysia; ^4^Department of Veterinary Preclinical Sciences, Faculty of Veterinary Medicine, Universiti Putra Malaysia, 43400 Serdang, Selangor, Malaysia

## Abstract

This study investigated the healing effects of topical application of zerumbone, a well-known anti-inflammatory compounds loaded on nanostructured lipid carrier gel (Carbopol 940) (ZER-NLCG) on excisional wounds in streptozotocin-induced diabetic rats. Diabetic rats with inflicted superficial skin wound were topically treated with ZER-NLCG, empty NLCG, and silver sulfadiazine cream (SSDC) once daily for 21 days. Wound tissue samples were analyzed for proinflammatory cytokines, namely, interleukin-6 (IL-6), interleukin-1 *β* (IL-1*β*), and tumor necrosis factor-*α* (TNF-*α*), hydroxyproline contents, catalase, superoxide dismutase activities, and lipid peroxidation level, and were subjected to histopathological analysis, respectively. Among the treated groups, ZER-NLCG was the most effective at decreasing proinflammatory cytokine level and inflammatory cell infiltration while increasing antioxidant enzyme activities, hydroxyproline content, and granulation of wound tissues of diabetic rats. ZER-NLCG is a potent formulation for the enhancement of wound healing in diabetic rats through its anti-inflammatory, antioxidant, and tissue repair activities.

## 1. Introduction

Wound healing is a well-organized interplay between migration and proliferation of blood cells, endothelial cells, extracellular matrix production, fibroblasts, cytokines, growth factor release, and angiogenesis [1, 2]. Delayed wound healing is a major complication in diabetics, and this is principally associated with hyperglycemia, abnormalities in cytokine expressions, oxidative stress, and impaired neovascularization [3–5]. In fact, diabetic patients are prone to develop chronic nonhealing foot ulcers [6, 7].

Numerous phytochemicals have been shown to have wound healing properties [8]. These phytochemicals include curcumin, picroliv, and arnebin-1 [9], thymoquinone [10], and zerumbone [11]. The wound healing properties of phytochemicals are partly exerted through their natural antioxidant properties [12, 13]. Unfortunately, many phytochemicals are water-insoluble, which compromise their therapeutic potentials. Several carriers have been used to solubilize these compounds. These include cyclodextrins, chitosan, liposome nanoparticles, and nanostructured lipid carriers (NLC) [14, 15]. Nanostructured lipid carriers are second-generation lipid-based nanoparticles that are developed based on the solid lipid nanoparticles (SLN) [16]. Unlike SLN, which is primarily composed of solid lipids, the NLC has a core structure comprising of both solid and liquid lipids. The core of the NLCs has an imperfect matrix structure that increased space for accommodation of drug, drug load, and with less potential for drug expulsion than SLN [17–19].

Zerumbone is the major sesquiterpene phytochemical in the rhizome of edible ginger *Zingiber zerumbet* (L.) Smith [20]. Zerumbone possesses antioxidant, anti-inflammatory, antimicrobial, hepatoprotective, antinociceptive [21], and anticancer [22, 23] properties. Despite the known therapeutic applications, the effect of zerumbone on wound healing process is still not investigated. Zerumbone has been loaded in NLC and the complex has shown to have anticancer properties [24].

Therefore, the present study was undertaken to determine the wound healing effects of free and NLC-loaded zerumbone in a streptozotocin-induced diabetic rat model.

## 2. Materials and Methods

### 2.1. Chemicals and Reagents

Streptozotocin (STZ) and sodium citrate were purchased from Sigma-Aldrich (St. Louis, USA). Ketamine and xylazine were purchased from Troy Laboratories Pte. Ltd. (New South Wales, Australia). The assay kits used for the determination of proinflammatory, hydroxyprolin levels, and enzyme activities were supplied by Cusabio Biotech (Houston, USA), while the issue lysis buffer (tissue protein extraction) reagent was purchased from Thermo Scientific Ltd. (Massachusetts, USA).

### 2.2. Preparation of Zerumbone-Nanostructure Lipid Carrier (ZER-NLCG) Gel

Pure colourless zerumbone (ZER) crystal was extracted from fresh *Zingiber zerumbet* rhizome essential oils using steam distillation, and zerumbone-nanostructure lipid carrier (ZER-NLC) was prepared and characterized as described by Rahman et al. [25]. The ZER-NLC gel (ZER-NLCG) and NLC gel (NLCG) were prepared as described earlier [26]. Briefly, the ZER-NLC has the particle size of 52.68 ± 0.1 nm and polydispersity index of 0.29 ± 0.0041 nm. The zeta potential of this compound is recorded at a level of 25.03 ± 1.24 mV, while the drug entrapment value is approximately 99.03% *w*/*w*.

### 2.3. Experimental Animals

All studies were performed in accordance to the guidelines of the Institutional Animal Care and Use Committee (ACUC), University of Malaya (ISB/22/07/2013/SFA).

In the present study, healthy and normal glycaemic male Wistar albino rats weighing in between 200 to 250 g were used. Animals were housed in stainless-steel cages under controlled ambient temperature of 25 ± 2°C and 12 h light-dark cycle. Commercial rat/mouse pellet (Specialty Feeds, Western Australia) and water were provided ad libitum.

### 2.4. Streptozotocin-Induced Diabetes

The rats were weighed and their fasting blood glucose were determined before the induction of diabetes. Food and water were withhold from the rats for 18 hours, and then, they were induced with a single intraperitoneal injection of 65 mg/kg streptozotocin (Sigma-Aldrich, USA) dissolved in 0·05 M sodium citrate (pH 4.5) to develop diabetes mellitus condition. Two days after the induction, the development of hyperglycemia (blood glucose levels higher than 16.7 mmol/L) in the rats was confirmed by estimating the fasting blood glucose concentrations using the Glucocheck strip (Accu-Chek® Active, Roche Diagnostic GmbH, USA) [27].

### 2.5. Excision Wound Model

Full-thickness excision skin wounds were created in the diabetic rats as described previously [28]. Briefly, while under anesthesia with intramuscular injection of ketamine hydrochloride (25 mg/kg)-xylazine hydrochloride (10 mg/kg), the hair on the dorsal neck of diabetic and normal control rats were shaved and the exposed skin were cleaned with 70% ethanol. A circular full thickness wound of 8 mm diameter was created aseptically on the exposed skin of each rat using a sterile skin biopsy punch (Biopunch®, Fray Products Corp., New York, USA). The wounds were dabbed with cotton soaked in normal saline to stop the bleeding [29].

### 2.6. Animal Grouping and Treatment

Diabetic rats were divided into five groups (*n* = 6/group). Normal nondiabetic rat control was assigned as group 1. Untreated diabetic rats were assigned as group 2. In group 3 which is considered as positive control, rats were treated with 0.2 ml 1% *w*/*w* silver sulfadiazine cream (SSDC) (reference standard). In group 4, rats were topically treated with 0.2 ml of NLCG, whereas in group 5, rats were topically treated with 0.5 mg/ml ZER-NLCG. These treatments were given for 21 days.

### 2.7. Healing and Wound Contraction

The size of the open wound was determined with planimetry by tracing the wound with transparent sheets, and the total area was determined with graph papers. The rate of healing was calculated and expressed as percentage wound contraction on days 7, 14, and 21. The wounds were photographed. The rate of healing was calculated using the following formula [28].

(1) $$\begin{array}{c} \text{Wound closure }\left(\%\right)=\frac{A_{0}\left({\text{mm}}^{3}\right)–A_{t}\left({\text{mm}}^{3}\right)}{A_{0}\;\left({\text{mm}}^{3}\right)}\times 100, \end{array}$$

where *A*_0_ is wound area on day 0 and *A*_*t*_ is the wound area postwound infliction.

### 2.8. Biochemical Analysis

#### 2.8.1. Determination of Blood Glucose Levels

Approximately 0.1 ml of blood was collected from the tail vein of each rat. A glucometer was used to determine the blood glucose levels immediately before and postwound infliction on days 7, 14, and 21.

#### 2.8.2. Determination of Collagen Synthesis

The total collagen content in granulation tissue samples was estimated based on the hydroxyproline level. Wound tissue samples were homogenized in ice-cold tissue lysis buffer and centrifuged at 10,000 × *g* for 5 min, and the hydroxyproline contents in the supernatants were quantitatively determined by ELISA (CSB-E08838r Cusabio Biotech Co., Ltd., Wuhan, China).

#### 2.8.3. Determination of Levels of Proinflammatory Cytokines

Wound tissue samples were excised and wound lysates prepared using tissue lysis buffer with protease and phosphatase inhibitors and analyzed for the presence of immunoreactive IL-1*β*, lL-6, and TNF-*α* by using ELISA kits.

#### 2.8.4. Oxidative Stress Level Determination

Skin tissue samples were prepared according to the method described by Albaayit et al. [28]. The activities of catalase (CAT) and superoxide dismutase (SOD) and lipid peroxidation (LPO) were determined by using ELISA kits.

### 2.9. Histology

Skin wound tissues of the rats were removed immediately after sacrifice and fixed in 10% buffered formaldehyde solution pH 7.4, dehydrated through graded concentrations of 70 and 95% of absolute ethanol, cleared in xylene, and embedded in paraffin wax at the melting point of 56°C. The tissues were sectioned to 5 *μ*m thickness and stained with haematoxylin and eosin (H&E) and examined microscopically at 400x magnification. The wounds were histologically scored using method described by Atiba et al. [30]. Lesion parameters were scored according to their distribution in each microscopic field.

### 2.10. Statistical Analysis

Data were expressed as means ± standard deviation (SD). Comparisons between groups and between days were performed using one-way analysis of variance (ANOVA) using GraphPad InStat version 3.0 (2000) statistical software. The level of significance was set at *p* < 0.05.

## 3. Results

### 3.1. Blood Glucose Level

Mean blood glucose was found to be significantly stable at higher levels and in the diabetic control and NLCG-, SSDC-, and ZER-NLCG-treated diabetic rats compared to the nontreated normal rats over the 21-day period of the study (Table 1).

### 3.2. Zerumbone-Nanostructure Lipid Carrier Gel Enhanced Wound Closure

**Figure 1:**
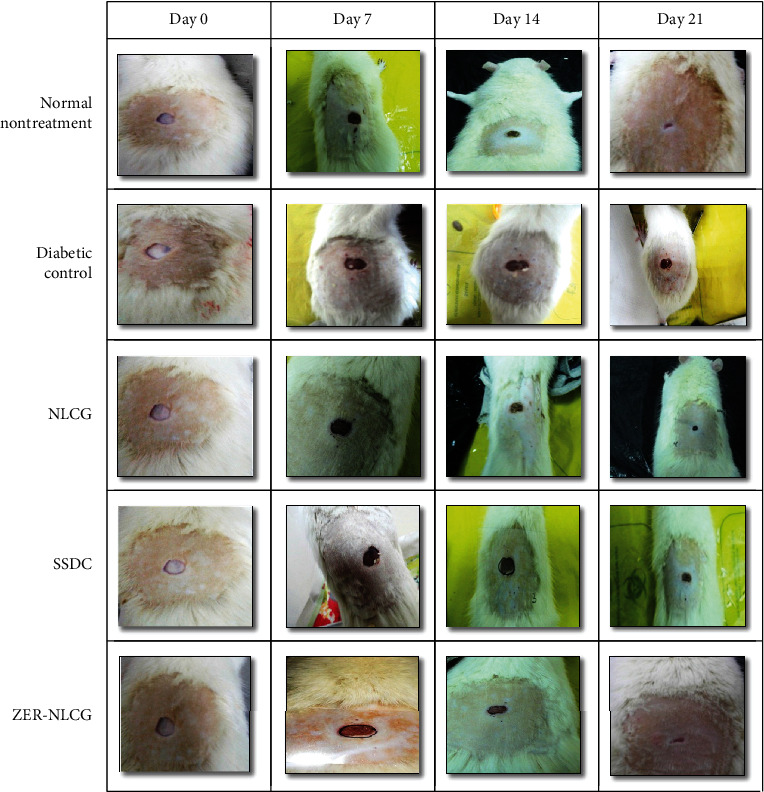
Wound healing in diabetic rats treated with NLCG, SSDC, and ZER-NLCG. ZER-NLCG produced the most best healing effect among treatment with wound contraction rate similar to that of nontreated normal rats.

**Figure 2:**
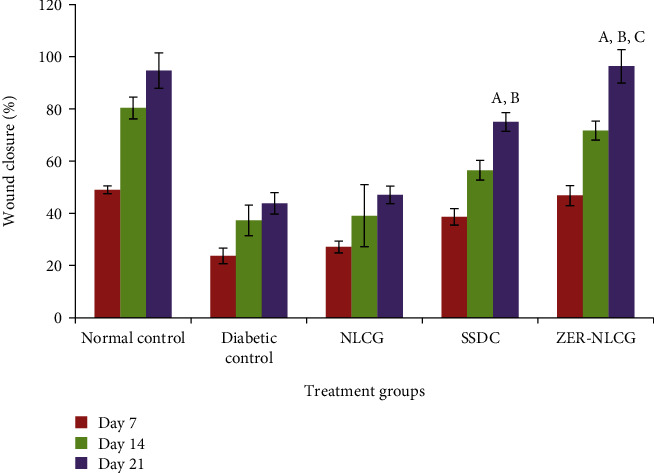
Wound contraction in diabetic rats treated with NLCG, SSDC, and ZER-NCLG. ZER-NLCG was most effective among treatments at acceleration wound closure in diabetic rats. Means significantly different from those of diabetic control^a^, NLCG-treated group^b^, and SSDC-treated group^c^ at *p* < 0.01. Values are expressed as mean ± SD.

Diabetic rats treated with ZER-NLCG showed accelerated and significant wound closure with almost complete epithelialization and healing by day 21 in comparison with the nontreated diabetic control or the SSDC-treated diabetic rats (Figure 1). The rate of wound healing in diabetic rats treated with ZER-NLCG is similar to that of the normal rats. The quantitative assessment of wound healing of the treated diabetic rats is shown in Figure 2. The most significant wound contraction in order of percentage was as follows: ZER-NLCG, followed by SSDC and NLCG treatment. By day 21, wounds treated with ZER-NLCG had contracted by 96% compared to 43.7% in the nontreated normal rats.

### 3.3. Histological Evaluation of Wound Tissues

**Figure 3:**
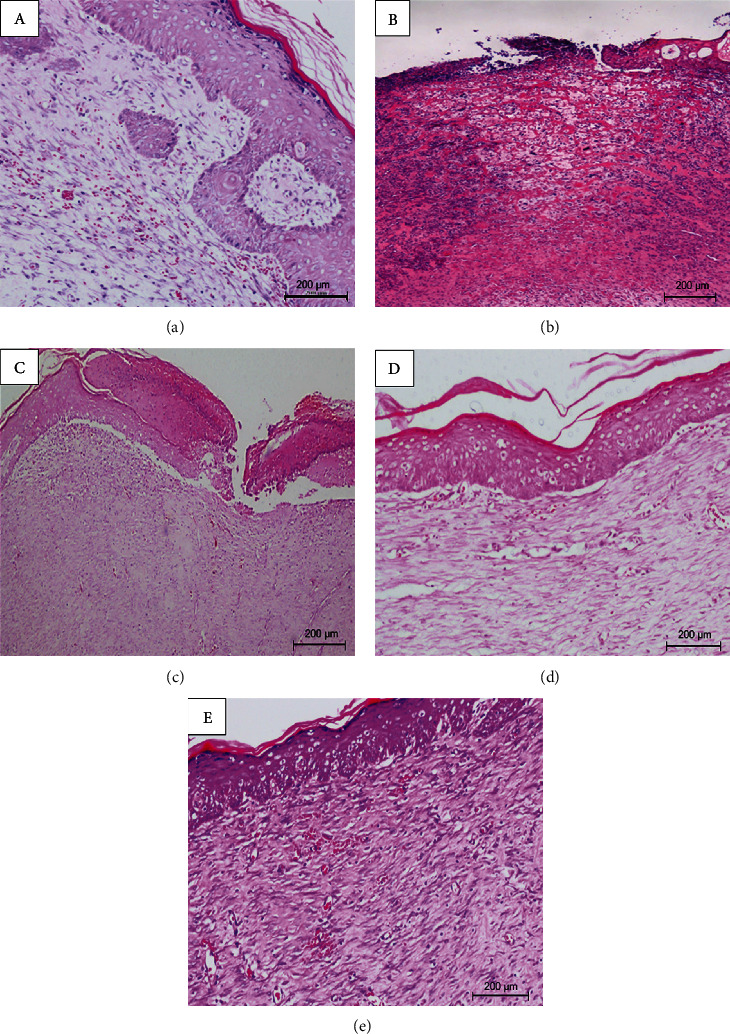
Skin wound tissue of diabetic rats treated with (c) NLCG, (d) SSDC, and (e) ZER-NLCG after 21 days. (a) Nontreated normal wound tissue and (b) nontreated diabetic wound tissue.

Nontreated normal and diabetic rats treated with SSDC and ZER-NLCG showed significant epithelialization in the epidermal layer and connective tissue after 21 days, which was evident in the in NLCG-treated and nontreated diabetic rats (Figure 3) (Table 2). The treated diabetic rats also showed a well-grown mature granulation tissue rich with newly formed capillaries and collagen fibers. Nontreated diabetic rats showed incomplete and mild granulation tissue in the wounds. Based on the histological changes, wound tissues of nontreated diabetic showed much slower healing than the treated diabetic rats. The effect of ZER-NLCG on wound healing was essentially similar to that of SSDC. Nontreated diabetic rats showed much slower healing than the treated diabetic rats. The effect of ZER-NLCG on wound healing was essentially similar to that of SSDC.

### 3.4. Hydroxyproline

**Figure 4:**
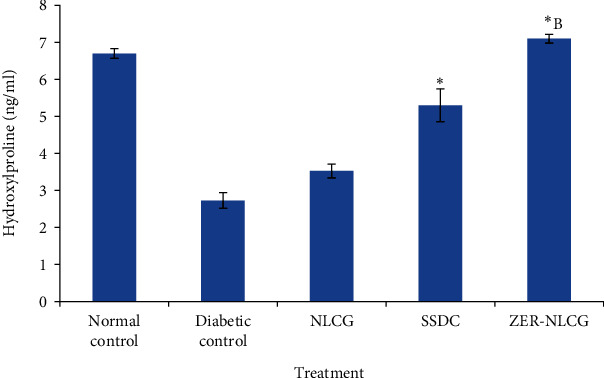
Hydroxyproline concentration in skin biopsies of diabetic rats treated with NLCG, SSDC, and ZER-NLCG. The concentrations of hydroxyproline were significantly lower in nontreated diabetic than normal rat skin. Among treatments, ZER-NLCG was the most effective in increasing hydroxyproline levels in the diabetic rat skin. Means significantly different from those of (diabetic (nontreatment) control and NLCG-treated rats)^∗^ and SSDC-treated rats^∗^^B^ at *p* < 0.05.

The concentration of hydroxyproline was significantly lower (*p* < 0.05) than in the skin of nontreated diabetic than normal rats (Figure 4). Treatments with NLCG, SSDC, and ZER-NLCG increased the level of hydroxyproline in the skin of the diabetic rats. ZER-NLCG was the most effective, among treatments, at elevating wound tissue hydroxyproline in diabetic rats to a level similar to that observed in the normal rats.

### 3.5. Proinflammatory Cytokines

**Figure 5:**
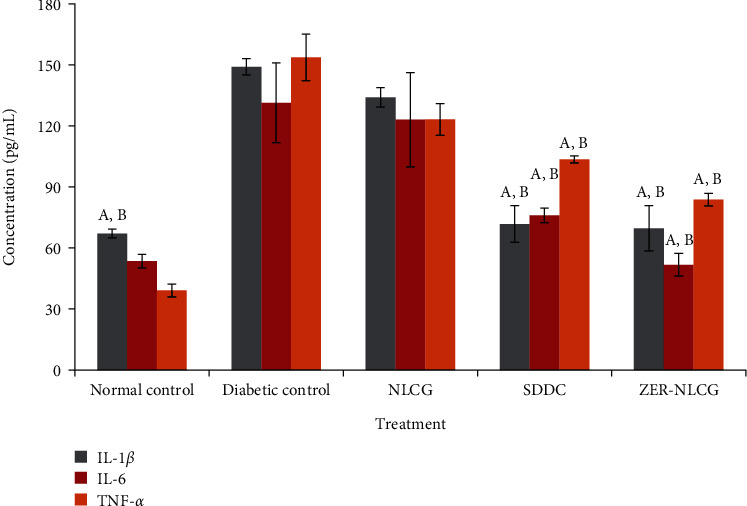
Cytokine concentrations in skin biopsies of rats treated with NLCG, SSDC, and ZER-NLCG. The concentrations of tumor necrosis factor-*α* (TNF-*α*), interleukin-1*β* (IL-1*β*), and interleukin-6 (IL-6) were significantly higher in nontreated diabetic than normal rat skin. Among treatments, ZER-NLCG was the most effective in reducing cytokine levels in the diabetic rat skin. Means significantly different from those of diabetic (nontreatment) control rats^a^ and NLCG-treated rats^b^ at *p* < 0.05.

Figure 5 presents the effect of treatments on the IL1-*β*, IL-6, and TNF-*α* levels in the granulation tissues of control after 21 days. Treatment with NLCG, SSDC, and ZER-NLCG lowered the concentrations of cytokines in the diabetic rat skin tissue. However, significant decreases (*p* < 0.001) were only observed in rats treated with SSDC and ZER-NLCG. ZER-NLCG was more effective than SSDC at reducing skin tissue cytokine levels.

### 3.6. Oxidative Stress Markers in Skin Tissue

**Figure 6:**
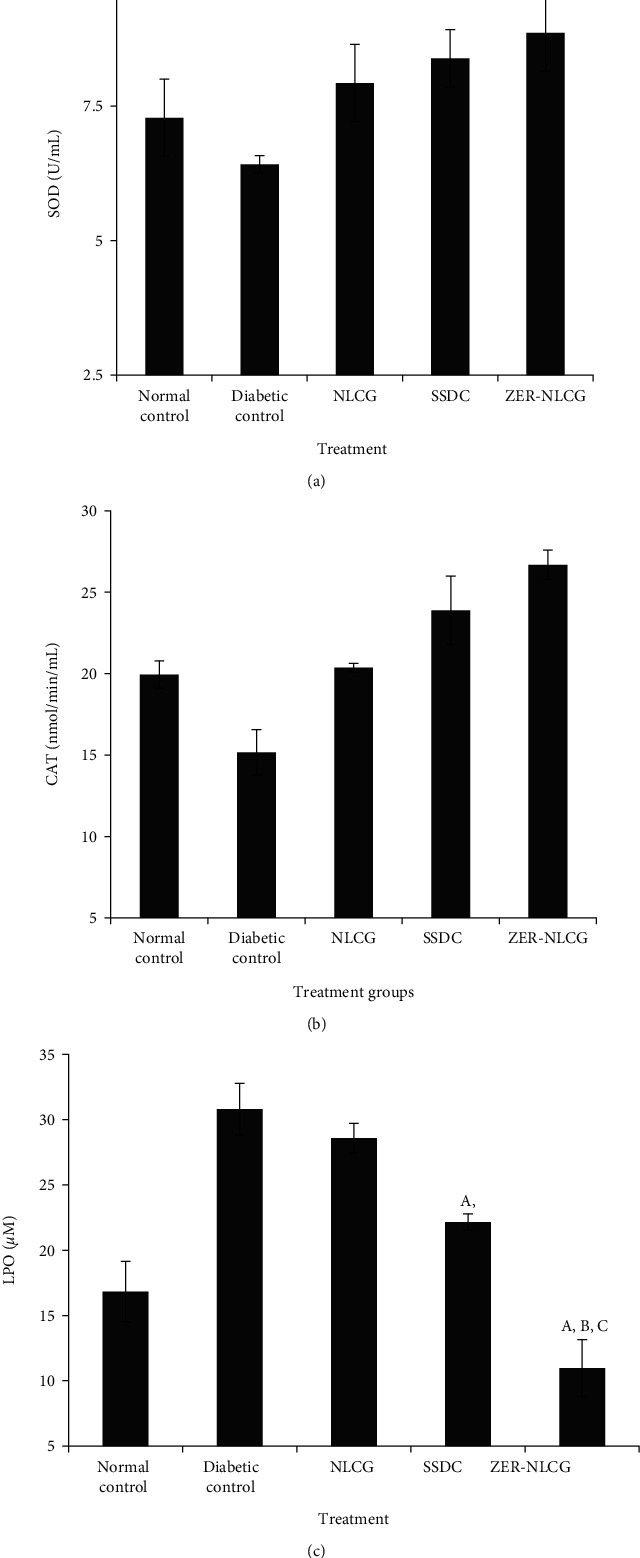
(a) Superoxide dismutase, (b) catalase, (c) glutathione peroxidase, and (d) lipid peroxidase (LPO) activities in skin biopsies of rats treated with NLCG, SSDC, and ZER-NLCG. Treatment generally increased SOD and CAT and decreased LPO activities. ZER-NLCG produced the most significant effect on the level of the enzymes. ^A^Means significantly different from those of diabetic (nontreatment) control rats^A^, NLCG-treated rats^B^, and SSDC-treated rats^C^ at *p* < 0.05.

Treatment generally increased SOD and CAT and decreased LPO activities (Figure 6). The SOD and CAT were highest while LPO activities were lowest in wound tissues of rats treated with ZER-NLCG.

## 4. Discussion

Repair of tissue injuries is defined by several stages: coagulation and hemostasis, inflammation, proliferation, and wound modeling with scar formation. Histological changes during wound healing begin to become evident at the proliferation stage. The proliferative stage is characterized by fibroblast migration and deposition of new extracellular matrix, angiogenesis, tissue granulation, and collagen synthesis [1, 26]. Wound healing in diabetes is usually chronic and this is the result of disruption in one or more of the healing stages. One the main causes of impaired and delayed wound healing in diabetic patients is hyperglycemia, which hampers fibroblast proliferation and decreases collagen production and impairs chemotaxis and phagocytosis [31]. In our study, nontreated diabetic rats showed incomplete and mild granulation in the wound tissues, suggesting a much slower wound healing process. Treatment with ZER-NLCG improved wound healing by prompting epithelial and keratin proliferation and epithelial differentiation. In the ZER-NLCG-treated rats, complete wound closure was observed by 21 days of treatment. This finding is supported by study from Gourishetti and colleague who indicates that the use of Sesamol-loaded PLGA nanosuspension accelerates wound healing and 90% wound closure was observed on day 15 of treatment [32].

There is ongoing search for new and cheaper therapeutic compounds from natural sources that could be used to heal wounds [33–39]. Among these compounds is zerumbone from the essential oil of the rhizomes of *Zingiber zerumbet* [40]. In pure crystalline form, zerumbone is water-insoluble due to which it has poor bioavailability and distribution in the body thus decreases its therapeutic potential. Recently, it was reported that zerumbone solubility increases upon loading into NLC [41]. The loading of zerumbone in NLC improves the biodistribution and effectiveness of the complex, allowing for parentally application of otherwise a water-insoluble compound.

Wound size and contraction are parameters used to evaluate healing of injured tissues [42–44]. In our study, wound contraction is determined by the percentage reduction in size of postwound infliction. The application of ZER-NLCG had hastened wound healing in diabetic rats through the influence of its active principle, zerumbone. Diabetic rats showed better wound healing after ZER-NLCG than SSDC treatment. ZER-NLCG decreased the infiltration inflammatory cells and increased intensity of collagen maturation and distribution in the wound tissues. The study showed that ZER-NLCG healed wounds without scarring.

Hydroxyproline is a major component of collagen. Hydroxyproline functions to stabilize the triple helix structure of collagen. The molecule is also used as an indicator of collagen content in tissues [45]. In wound healing, collagen formation peaks and under optimal condition epithelialization occurs after 22 to 48 postinjury [46]. ZER-NLCG significantly (*p* < 0.001) increased wound tissue hydroxyproline level (Figure 4), suggesting that there were increased synthesis and deposition of collagen in the wound tissues with the treatment. Based on the hydroxyproline level, ZER-NLCG was more efficacious at healing wounds in diabetic rats than SSDC.

Inflammation plays a vital role in normal wound healing process [47–49]. Inflammation delays healing of injured tissues and can cause increased scarring [50, 51]. In diabetes, tissue injuries are associated with elevated levels of proinflammatory cytokines [52] that affect the recruitment of various inflammatory cells and function of fibroblast and keratinocyte, production of extracellular matrix, and angiogenesis and impaired wound healing [5, 53]. In diabetic rats, ZER-NLCG more than SSDC significantly downregulated the proinflammatory cytokines, TNF-*α*, IL-1*β*, and IL-6. These findings highlight that the inhibition inflammatory response is one of the many facets of the wound healing effects of ZER-NLCG.

Oxidative stress in tissues is the result of the imbalance between the level of reactive oxygen species (ROS) and the ability of antioxidative mechanism to remove the ROS [54, 55]. While low tissue ROS levels initiate, high levels hinder the normal process of tissue healing [56, 57]. The tissue ROS level is high in diabetes due to decrease or/and increase in catalase (CAT), superoxide dismutase (SOD), and glutathione peroxidase (GSH-Px) production. The constant state of hyperglycemia in diabetes also increases the production of free radicals via the oxidation of glucose and the glycation of nonenzymatic proteins [58]. ZER-NLCG exerts antioxidant effects by increasing SOD and CAT and decreasing LPO activities. These effects of ZER-NLCG prevented inflammation and oxidative damage and promoted healing of wounds in diabetic rats. Previous study has also indicate that sustained drug release as in our study using ZER-NCLG are able to reduce the level of reactive oxygen species (ROS) thus increasing its antioxidant activity and promotes a much faster wound healing [59, 60].

## 5. Conclusion

Topical applications of ZER-NLCG might effectively improve wound healing in streptozotocin-induced diabetic rats by suppressing production of proinflammatory cytokines, reducing inflammatory response, increasing collagen deposition, and reducing the effect of ROS by stimulating production of antioxidant enzymes without scarring.

## Figures and Tables

**Table 1:** Blood glucose concentration in diabetic rats treated with NLCG, SSDC, and ZER-NLCG.

Treatment Groups (*n* = 6)	Blood glucose (mmol/l)			
	Days postwound infliction			
	0	8	14	21
Normal (nontreated)	5.23^a^ ± 0.07	5.35^a^ ± 0.12	5.26^a^ ± 0.18	5.30^a^ ± 0.14
Diabetic (nontreated)	18.63^b^ ± 0.55	18.73^b^ ± 1.32	19.07^b^ ± 0.98	19.03^b^ ± 1.99
NLCG	19.43^b^ ± 1.68	19.30^b^ ± 2.30	19.37^b^ ± 1.13	19.78^b^ ± 2.03
SSDC	19.44^b^ ± 1.52	19.54^b^ ± 1.97	18.73^b^ ± 0.84	17.08^b^ ± 2.09
ZER-NLCG	20.33^b^±1.46	20.15^b^ ± 1.40	18.83^b^ ± 2.20	20.58^b^ ± 1.86

^a,b^Means with different superscript within columns are statistically significant (*p* < 0.05) different from the normal values.

**Table 2:** Histological changes in wound tissues of diabetic rats treated with NLCG, SSDC, and ZER-NLCG.

Parameter	Lesion score (%)				
	Normal control	Diabetic control	NLCG	SSDC	ZER-NLCG
Epidermal wound closure	87.7^a^ ± 3.6	0.7^b^ ± 0.9	51.7^a^ ± 12.6	100^c^ ± 0	100^c^ ± 0
Epithelial proliferation and differentiation	48.3^a^ ± 10.2	0.3^b^ ± 1.9	2.3^b^ ± 1.1	83.3^c^ ± 2.4	86.7^c^ ± 4.7
Epithelial ballooning	13.0^a^ ± 15.8	53.3^b^ ± 4.7	37.0^a^ ± 18.8	16.6^a^ ± 2.4	10.0^c^ ± 0.0
Keratin proliferation	56.6^a^ ± 13.1	0	0	86.6^b^ ± 4.7	88.0^b^ ± 2.4
Edema	9.0^a^ ± 5.4	40.0^b^ ± 4.1	47.0^b^ ± 3.1	0	0
Epidermal PMN cell infiltration	10.7^a^ ± 3.3	85.0^b^ ± 4.1	73.0^b^ ± 3.1	3.7^c^ ± 0.9	4.7^b^ ± 3.8
Dermal PMN cell infiltration	23.3^a^ ± 8.5	75.0^b^ ± 4.1	71.0^b^ ± 3.1	26.6^c^ ± 16.5	8.3^c^ ± 2.4
Neovascularization	75.0^a^ ± 4.1	65.0^b^ ± 4.1	80.0^b^ ± 4.1	75.0^a^ ± 4.1	75.0^a^ ± 4.1
Fibroblast proliferation	61.7^a^ ± 8.5	75.0^b^ ± 4.1	65.0^b^ ± 4.0	81.6^b^ ± 6.2	83.3^b^ ± 6.2
Collagen proliferation index	75.0^a^ ± 4.1	8.3^b^ ± 2.6	3.3^b^ ± 2.3	71.7^c^ ± 6.2	70.0^c^ ± 4.1

Values are mean ± std dev. ^a,b,c^Means within row with different superscripts are significantly different at *p* < 0.05. PMN = polymorphonuclear cell.

## Data Availability

Data used to support the findings of this study are included within the article.
